# Supplementary material for: A Live Cell Protein Complementation Assay for ORFeome-Wide Probing of Human HOX Interactomes
Source: Cells. 2023 Jan 3;12(1):200. doi: 10.3390/cells12010200 (PMC9818449; doi:10.3390/cells12010200)
Supplement: Supplementary file 1 [file cells-12-00200-s001.zip › Figures S1-S11.pdf]

A live cell protein complementation assay for ORFeome-wide probing of human HOX interactomes

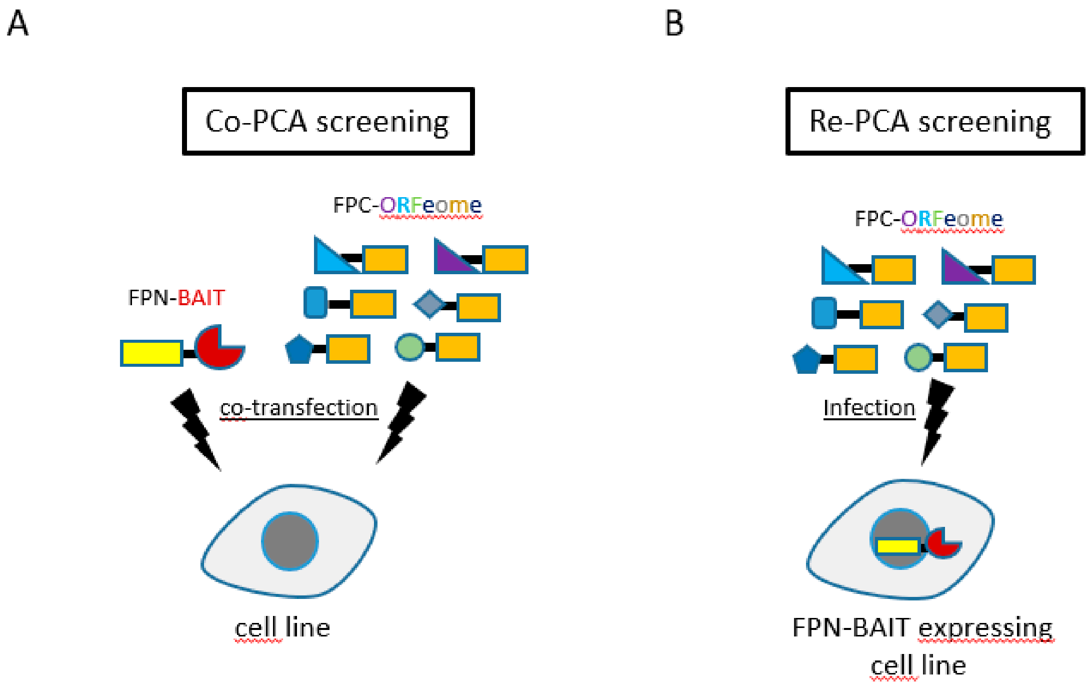

Supplementary Figure S1. Application of PCA-based strategies for large-scale interaction screens in living cells. The co-transfection PCA-based screening strategy (Co-PCA) relies on the transitory expression of the FPN-fusion bait protein and the FPC-fusion human ORFeome library upon co-transfection in the cell line (Remy & Michnick, 2004; Berendzen, K.W. et al., 2012) (B). The retrovirus PCA-based screening strategy (Re-PCA) relies on the use of a cell line stably expressing the FPN-fusion bait protein and infected by retroviruses containing the FPC-prey human ORFeome library that is artificially expressed with a constitutive promoter (Lee *et al*, 2011; Simon E. Coopera, 2015) (D). A first generation of the Re-PCA screening strategy was based on random genomic insertion of retroviruses encoding the FPC in the three possible open reading (Ding *et al*, 2006).

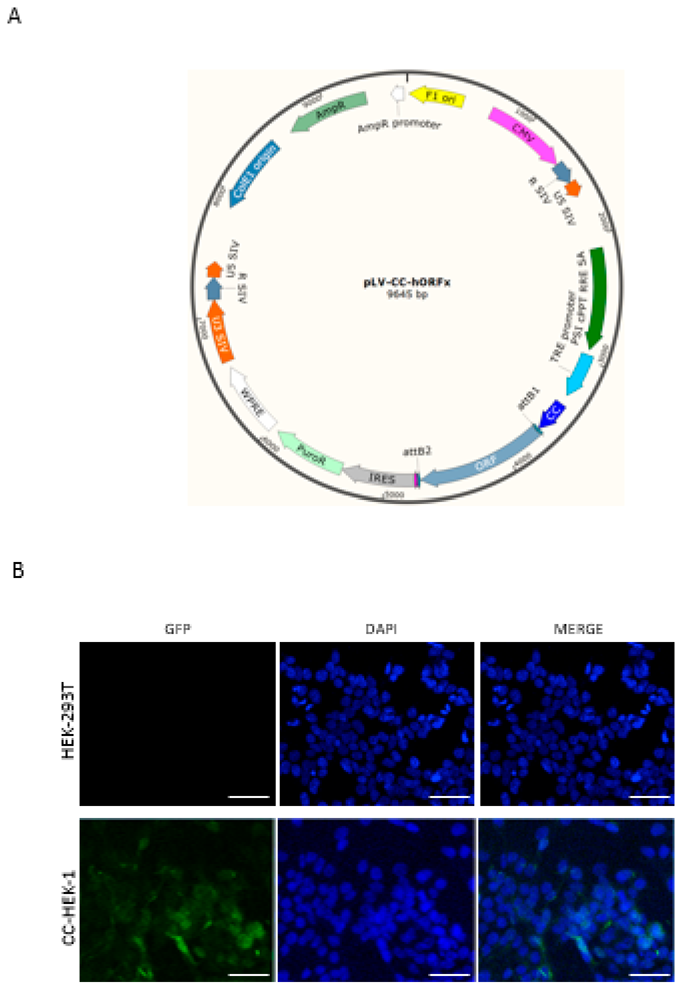

Supplementary Figure S2. Map of the lentiviral expression vector and basal expression level of the CC-ORF constructs in the established CC-HEK cell line. A. A lentiviral destination vector (pLV-CC-Gateway) was generated to clone the human ORFeome library in frame with the C-terminal fragment of Cerulean (CC), leading to the pLV-CC-ORF vector. The expression of the CC-ORF is under the control of a first-generation TRE promoter. This system (Tet-Off) requires the co-expression of a Tet transactivator (tTA) for full activation of the promoter. **B.** The basal activity of the TRE promoter (without adding Dox and the tTA-coding vector) is sufficient to detect the expression of the inserted CC-ORFs in the CC-HEK1 cell population. Control with the original HEK-293T cell lines leads no staining. Immunostaining was performed with an anti-GFP recognizing the CC fragment (green). DAPI (blue) stains for nuclei. Scale bar = 50 µm.

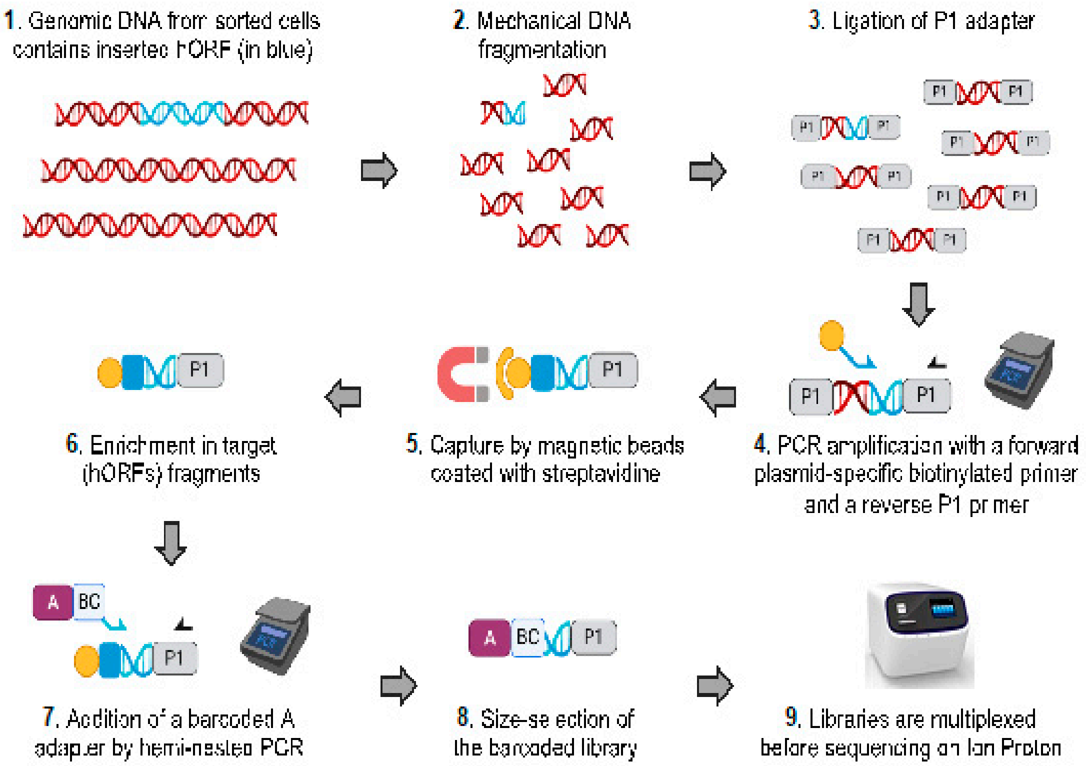

Supplementary Figure S3. Schematic of the ORF Capture-Sequencing method.

Libraries were constructed using our own designed proprietary protocol in order to enrich in sequences covering the beginning of all the CC-ORFs inserted in the genome. Roughly, the DNA extracted from sorted cells (1) is fragmented using the Covaris S220 Ultrasonicator (2). Then, the Ion adapter P1 is non-directionally ligated to both ends of the DNA fragments according to the standard blunt-end ligation protocol for Ion Torrent libraries (3). Because the P1 libraries molecules containing hORF sequences are underrepresented, an enrichment by a first PCR is performed by using a biotinylated forward primer located on the plasmid sequence upstream the hORFs and a reverse primer located at the end of the P1 adapter (4). Biotinylated PCR fragments, containing now at each end the beginning of the hORFs and the P1 adapter respectively, are then captured using streptavidin-coupled magnetic beads (5, 6). These molecules are used as a template for the final hemi-nested PCR amplification using a forward fusion primer containing a barcoded Ion A adapter and the same reverse primer as before (7). The hemi-nested PCR reinforces the specificity of the fragments to be sequenced since the primer

fused to A and the barcode is located closer to the hORF beginning on the plasmid. After a size selection using SPRI beads to meet Ion Torrent requirements (8), the qualified and quantified barcoded libraries are multiplexed in an equimolar manner and sequenced on the Ion Proton sequencer using a P1 chip following the manufacturer's recommendations (9).

A

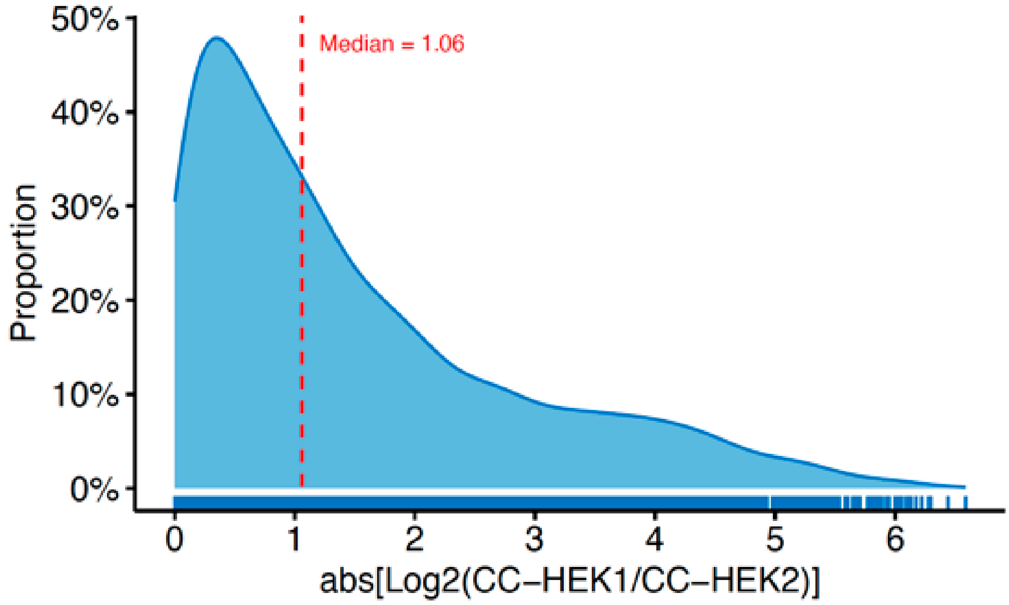

B

	3222 genes <3		1135 genes 3< <10		648 genes >10	
	unique	common	unique	common	unique	common
A1	117 genes		66 genes		86 genes	
	31 (27%)	86 (73%)	38 (57%)	28 (43%)	64 (74%)	22 (26%)
A2	112 genes		62 genes		83 genes	
	39 (35%)	73 (65%)	36 (58%)	26 (42%)	59 (71%)	24 (29%)
C6	107 genes		66 genes		79 genes	
	51 (24%)	56 (76%)	38 (58%)	28 (42%)	57 (72%)	22 (28%)
A7	111 genes		68 genes		73 genes	
	27 (24%)	84 (76%)	36 (53%)	32 (47%)	46 (63%)	27 (37%)
C8	99 genes		60 genes		78 genes	
	59 (60%)	40 (40%)	38 (63%)	22 (37%)	62 (80%)	16 (20%)
A9	111 genes		73 genes		86 genes	
	41 (37%)	70 (63%)	46 (63%)	27 (37%)	68 (79%)	18 (21%)
D10	97 genes		66 genes		81 genes	
	50 (52%)	47 (48%)	37 (56%)	29 (44%)	66 (82%)	15 (18%)
B13	100 genes		47 genes		93 genes	
	38 (38%)	62 (62%)	25 (53%)	22 (47%)	73 (79%)	20 (21%)

Supplementary Figure S4. Differential frequency counts of commonly integrated CC-ORFs in the CC-HEK1 and CC-HEK2 cell lines. A. Graph showing the repartition of inserted CC-ORFs in CC-HEK1 and CC-HEK2 cell lines. The x-axis is log2-transformed for the ratio of gene counts between the two CC-HEK cell lines. The proportion in total is depicted along the y-axis. The median indicates that 50% of inserted CC-ORFs have a difference bigger or smaller

than log2FC=1.06 (around 2-fold). **B.** Table indicating the proportion of unique (specific of CC-HEK1 or CC-HEK2) or common positive interactions for the different HOX among the 5005 common integrated genes, and depending on the variation of the insertion frequency between the two cell lines (less than 3 times difference; between 3- and 10-times difference, more than 10 times difference).

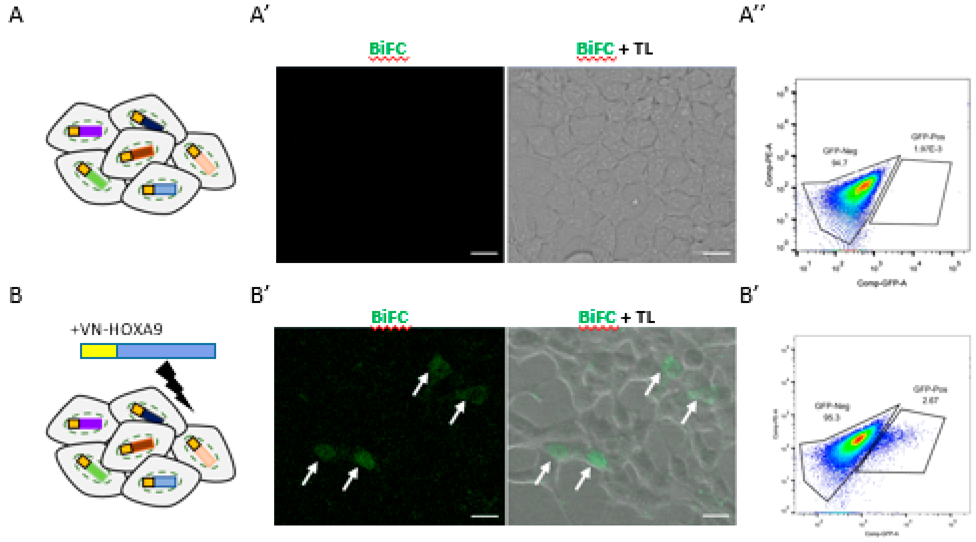

Supplementary Figure S5. Testing for specific BiFC signal and FACS gate for BiFC-positive cell population. A. Schematic representation of the cold (non-transfected) CC-HEK cell line. **A'.** Illustrative confocal picture of non-transfected cells upon excitation wave length for BiFC. Transmission light capture shows the cells. **A".** FACs gates for the GFP channel. No fluorescent signal can be observed in the cold/non-transfected CC-HEK cell population. **B.** Schematic representation of VN-HOX9 transfection in the CC-HEK cell line. **B'.** Illustrative confocal picture of transfected cells upon excitation wave length for BiFC. Fluorescent cells are indicated (white arrows). Transmission light capture shows the cells. **B".** FACs gates for the GFP channel. Fluorescent signals can be observed in the CC-HEK cell lines upon transfection of VN-HOXA9. Compared to A", the specific GFP-positive population can be isolated from the negative/non-fluorescent cells. This population represents 2.67% of the total cell population. A similar percentage of fluorescent cells (between 1,5% and 3%) was obtained with the different HOX constructs.

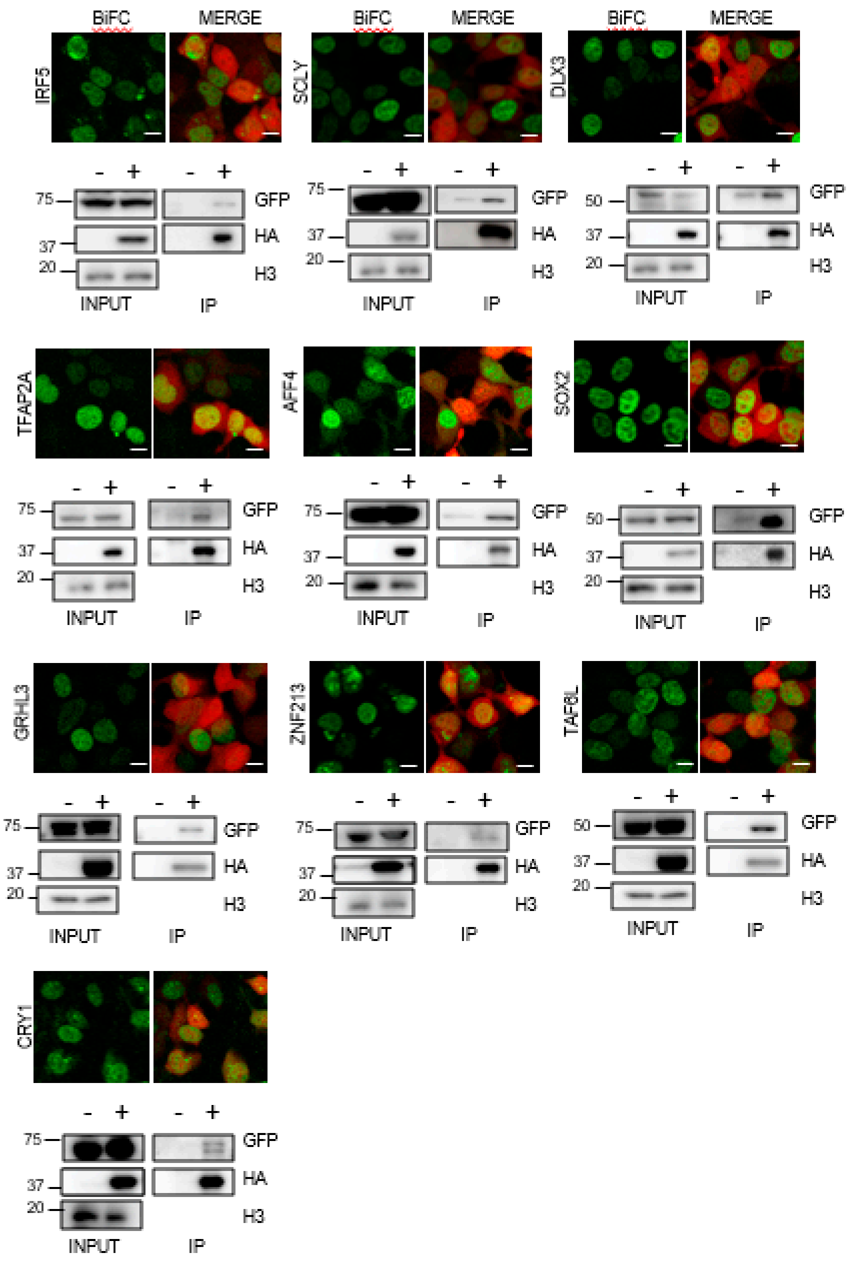

Supplementary Figure S6. Individual validation of positive HOXA9 interactions selected from the BiFC screen in the two CC-HEK cell lines. Illustrative confocal pictures of BiFC and co-immunoprecipitation (Co-IP) in HEK293T cells between HOXA9 and the selected candidates, as indicated. Confocal and western blot pictures are illustrative of at least two independent biological replicates. For BiFC experiments, the mCherry reporter (red, merge panels) is indicative of the transfection efficiency. Note the various intra-cellular BiFC profiles with different candidates. When two candidates were of different sizes, the protein extracts were loaded on the same gel (for DLX3/TFAP2A and GRHL3/TAF6L). Scale bar,

10µm. Co-IP was performed with anti-HA recognizing HA-HOXA9 and the CC-ORF was revealed with anti-GFP. Staining with Histone H3 antibody validates the correct protein extraction in each condition. “+“ and “-” respectively denote the co-transfection or not of HA-HOXA9 with the CC-ORF construct. The protein size scale is indicated on the left side (KDa).

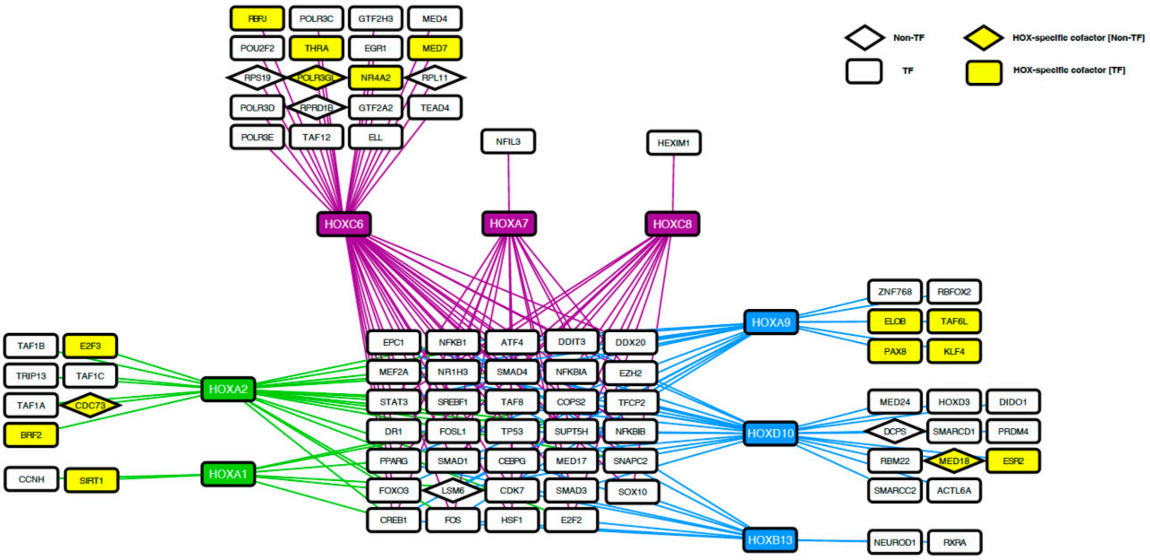

Supplementary Figure S7. Interactome of the enriched RNA-PolII dependent transcription function found in HOX BiFC screens. Transcription factors are boxed in a rectangle whereas non transcription factor partners are boxed in a rhomb. Yellow-colored boxes represent specific interactors of one HOX protein. Anterior HOX are in green, central HOX are in purple and posterior HOX are in blue.

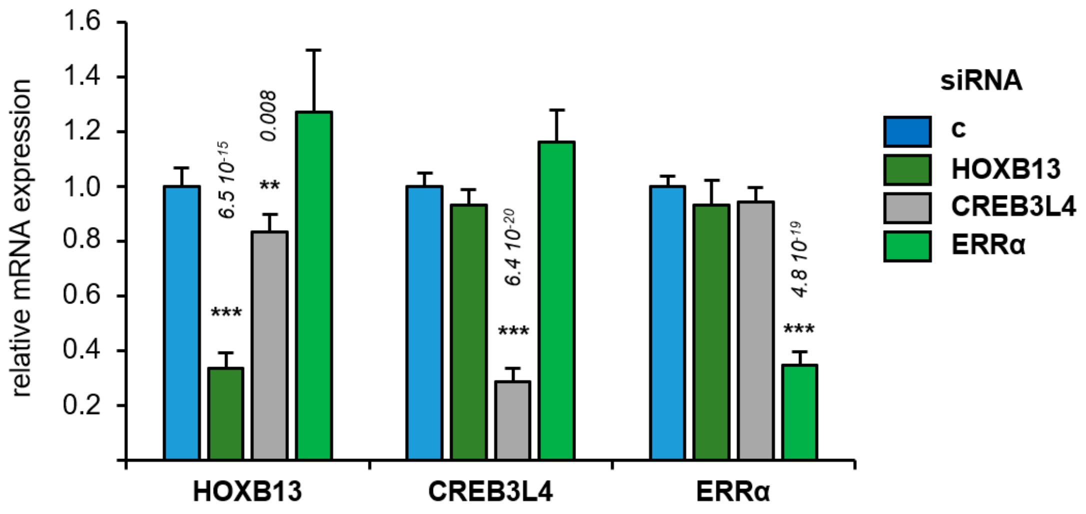

Supplementary Figure S8. Expression of *HOXB13*, *CREB3L4* and *ERRα* 48h post-transfection of the indicated siRNAs in PC-3 cells. Each siRNA significantly reduces the expression of its corresponding gene as evaluated by rt-qPCR. Results were obtained from eight independent experiments. Bars represent mean +/- sem. **: p<0.01, ***: p<0.005. t-test values (relative to control) are shown for significantly different points.

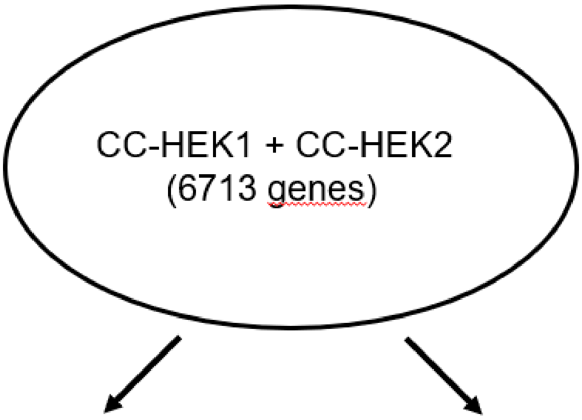

	Positive interactions in either CC-HEK1 or CC-HEK2 (log2FC≥6)	Common Positive interactions in CC-HEK1 and CC-HEK2 (log2FC≥1,5)
HOXA1	226	137 (38%)
HOXA2	258	123 (32%)
HOXC6	347	106 (23%)
HOXA7	218	143 (40%)
HOXC8	318	78 (20%)
HOXA9	298	115 (28%)
HOXD10	323	91 (22%)
HOXB13	227	104 (31%)

Supplementary Figure S9. Reproducibility of positive interactions among the 5005 commonly integrated CC-ORFs in the CC-HEK-1 and CC-HEK-2 cell lines. The third column is indicative of the percentage of reproducibility (% of commonly integrated CC-ORFs that were positive in both CC-HEK1 and CC-HEK2 cell line screens).

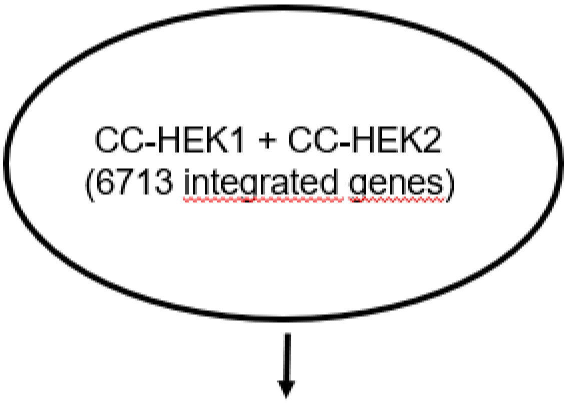

Positive interactions with all tested HOX proteins

Official_sym	ORF_size	DbTF_stat
ANKRD49	720	0
**CREB3L2**	744	1
**DDIT3**	510	1
HTATIP2	729	0
**ILF3**	2121	1
MTA1	768	0
**PSMC5**	1221	1
SSBP4	1158	0
TCF25	2031	0
**ZNF85**	168	1
**GRHL3**	1668	1
**RARB**	1347	1
**SUPT5H**	3264	1
**PER1**	885	1
NOL3	627	0

Supplementary Figure S10. List of interactors that are positive with all tested HOX proteins. All these interactors are nuclear proteins. DNA-binding-domain containing TFs are highlighted (bolded).

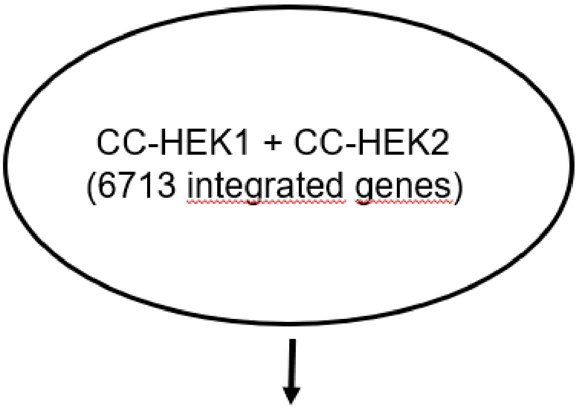

HOX proteins	Known cofactors (Biogrid)	Present in the CC-HEK Cell lines	Positive in the Cell-PCA screen	%
HOXA1	365	254	71	28
HOXA2	5	4	0	/
HOXC6	14	4	1	/
HOXA7	25	22	4	18
HOXC8	119	98	29	30
HOXA9	25	20	7	35
HOXD10	14	11	2	18
HOXB13	16	10	5	50

Supplementary Figure S11. Comparison of Hox positive interactions between Cell-PCA and the Biogrid database. Only genes corresponding to integrated CC-ORFs in the CC-HEK cell lines have been considered for the comparison. Paralogs and isoforms have also been considered to increase the number of genes that could be analyzed.
